# Protein intake and risk of urolithiasis and kidney diseases: an umbrella review of systematic reviews for the evidence-based guideline of the German Nutrition Society

**DOI:** 10.1007/s00394-023-03143-7

**Published:** 2023-05-03

**Authors:** Thomas Remer, Nicole Kalotai, Anna M. Amini, Andreas Lehmann, Annemarie Schmidt, Heike A. Bischoff-Ferrari, Sarah Egert, Sabine Ellinger, Anja Kroke, Tilman Kühn, Stefan Lorkowski, Katharina Nimptsch, Lukas Schwingshackl, Armin Zittermann, Bernhard Watzl, Roswitha Siener, the German Nutrition Society

**Affiliations:** 1 DONALD Study Center Dortmund, Department of Nutritional Epidemiology, Institute of Nutrition and Food Science, University of Bonn, Heinstück 11, 44225 Dortmund, Germany; 2 German Nutrition Society, Bonn, Germany; 3 Department of Aging Medicine and Aging Research, University Hospital Zurich, University of Zurich, and City Hospital Zurich, Zurich, Switzerland; 4 Department of Nutrition and Food Science, Nutritional Physiology, University of Bonn, Bonn, Germany; 5 Department of Nutrition and Food Science, Human Nutrition, University of Bonn, Bonn, Germany; 6 Department of Nutritional, Food and Consumer Sciences, Fulda University of Applied Sciences, Fulda, Germany; 7 The Institute for Global Food Security, Queen’s University Belfast, Belfast, Northern Ireland UK; 8 Faculty of Medicine and University Hospital, Heidelberg Institute of Global Health (HIGH), Heidelberg, Germany; 9 Department of Nutritional Sciences, University of Vienna, Vienna, Austria; 10 Center for Public Health, Medical University of Vienna, Vienna, Austria; 11 Institute of Nutritional Sciences, Friedrich Schiller, University Jena, Jena, Germany; 12 Competence Cluster for Nutrition and Cardiovascular, Health (nutriCARD) Halle-Jena-Leipzig, Jena, Germany; 13 Molecular Epidemiology Research Group, Max Delbrück Center for Molecular Medicine (MDC) in the Helmholtz Association, Berlin, Germany; 14 Faculty of Medicine, Institute for Evidence in Medicine, Medical Center - University of Freiburg, Freiburg, Germany; 15 Clinic for Thoracic and Cardiovascular Surgery, Herz- Und Diabeteszentrum Nordrhein-Westfalen, Ruhr University Bochum, Bad Oeynhausen, Germany; 16 Department of Physiology and Biochemistry of Nutrition, Max Rubner-Institut, Karlsruhe, Germany; 17 Department of Urology, University Stone Center, University Hospital Bonn, Bonn, Germany

**Keywords:** Protein guideline, Umbrella review, Urolithiasis, GFR, Urinary albumin excretion, Kidney health

## Abstract

**Purpose:**

Changes in dietary protein intake metabolically affect kidney functions. However, knowledge on potential adverse consequences of long-term higher protein intake (HPI) for kidney health is lacking. To summarise and evaluate the available evidence for a relation between HPI and kidney diseases, an umbrella review of systematic reviews (SR) was conducted.

**Methods:**

PubMed, Embase and Cochrane Database of SRs published until 12/2022 were searched for the respective SRs with and without meta-analyses (MA) of randomised controlled trials or cohort studies. For assessments of methodological quality and of outcome-specific certainty of evidence, a modified version of AMSTAR 2 and the NutriGrade scoring tool were used, respectively. The overall certainty of evidence was assessed according to predefined criteria.

**Results:**

Six SRs with MA and three SRs without MA on various kidney-related outcomes were identified. Outcomes were chronic kidney disease, kidney stones and kidney function-related parameters: albuminuria, glomerular filtration rate, serum urea, urinary pH and urinary calcium excretion. Overall certainty of evidence was graded as ‘possible’ for stone risk not to be associated with HPI and albuminuria not to be elevated through HPI (above recommendations (> 0.8 g/kg body weight/day)) and graded as ‘probable’ or ‘possible’ for most other kidney function-related parameters to be physiologically increased with HPI.

**Conclusion:**

Changes of the assessed outcomes may have reflected mostly physiological (regulatory), but not pathometabolic responses to higher protein loads. For none of the outcomes, evidence was found that HPI does specifically trigger kidney stones or diseases. However, for potential recommendations long-term data, also over decades, are required.

**Supplementary Information:**

The online version contains supplementary material available at 10.1007/s00394-023-03143-7.

## Introduction

Dietary habits are known to metabolically affect functions of numerous organs, including those of the kidney. Although single nutritional factors appear not to be directly or strongly involved in kidney function decline, long-term nutritional habits can relevantly contribute to renal impairment by modulating risk factors for chronic kidney disease (CKD), such as hypertension, hyperglycaemia, or obesity [1]. Moreover, several nutritional factors are known to play a crucial role in kidney stone formation [2]. One major nutrient, i.e. protein, has been in the focus of renal nutritional research for many decades since high-protein intake (HPI) has been observed in different studies to raise the risk of renal death, to accelerate the onset of dialysis in patients with CKD, and to increase urinary risk factors for kidney stone formation [3, 4]. However, conclusive findings on potential long-term consequences of habitually eating high-protein diets for kidney health are lacking in subjects with normal renal function [5].

Among the various outcomes related to kidney function assessment, urinary albumin (or protein) excretion and glomerular filtration rate (GFR) are the most prominent. Along with these parameters, urine pH and urinary calcium excretion, serum concentrations of urea and uric acid are also frequently examined. Nevertheless, their specificity as indicators for kidney function decline and kidney disease development has not yet been clarified in a causal context with HPI.

The evidence-based guideline for protein intake of the German Nutrition Society addresses the key question of whether the dietary intake of protein with regard to quantitative and qualitative considerations affects the development of kidney diseases in the general adult population. The current analysis focuses on the effect of HPI (generally above 0.8 g/kg body weight (BW)/day (d)) [6]) on kidney diseases and renal function-related parameters.

## Methods

We conducted an umbrella review (PROSPERO: CRD42018082395) [7] following the methodology published by Kroke et al. [8]. All methodological steps were conducted independently by two authors. Any disagreements were resolved by discussion to achieve consensus.

### Literature search

The systematic literature search was conducted in PubMed, Embase and Cochrane Database of Systematic Reviews for systematic reviews (SRs) published between 02/2008 and 12/2022. The date 02/2008 originates from the decision to cover a 10-year period, i.e. the initial database search was conducted in 02/2018, and the last update was made in 12/2022. The search strategies are presented in Supplementary Material S1. In addition to the database search, reference lists of included SRs were screened. Broad overall search strategies encompassing a wide range of potential renal function-related parameters, kidney diseases, and kidney stones were applied to gather all functionally or potentially pathophysiologically relevant kidney outcomes.

### Literature selection

Titles and/or abstracts of retrieved studies were screened according to predefined inclusion and exclusion criteria [8] to identify potentially eligible SRs. The full texts of these records were retrieved and assessed for eligibility. It was tolerated that some of the primary studies were incorporated more than once into different SRs. The overlap of primary studies is shown in Supplementary Material S2.

SRs were included if they met the following criteria: (i) the study evaluated the association between protein intake and kidney function-related outcomes or kidney diseases; (ii) the study population was the general adult population including older adults and athletes; (iii) the study design was an SR with or without meta-analysis (MA) of prospective studies with human study participants, i.e. randomised controlled trials (RCTs), prospective cohort studies, case–cohort studies, or nested case–control studies; SRs also considering case–control studies were only included if prospective studies were predominant (> 50% of all studies); (iv) publication was written in English or German and (v) published between 02/2008 and 12/2022 [8].

### Data extraction

The following relevant data from each included SR were extracted into a standardised table: first author of the SR, year of publication, study type of relevant primary studies, study period of relevant primary studies, study population of relevant primary studies, range of protein intake if provided, intervention/exposure(s) of primary studies, outcome(s) investigated by primary studies, effect estimates including 95% CI, *p* values, heterogeneity estimates, and subgroup analyses.

### Assessment of methodological quality and outcome-specific certainty of evidence

To assess the methodological quality of included SRs, a modified version of the AMSTAR 2 (A Measurement Tool to Assess Systematic Reviews 2) tool [9] was used (Supplementary Material S3), and the modifications are described in detail in our methodological protocol [8]. This version of AMSTAR 2 contains 14 items that evaluate the methodological quality of the SR. SRs were rated on a scale from high quality to critically low quality according to the existence of critical and non-critical methodological weaknesses. SRs graded as ‘critically low’ by AMSTAR 2 were excluded from the rating of the overall certainty of evidence.

The outcome-specific certainty of evidence of included SRs with and without MA was assessed using the NutriGrade scoring tool [10] (Supplementary Material S4). NutriGrade aims to assess the certainty of evidence of an association or effect between different dietary factors and outcomes, taking into account nutrition research-specific requirements not considered by other tools. The NutriGrade scoring tool utilises a numerical scoring system and comprises seven items for SRs with MA of RCTs and eight items for MA of cohort studies. Based on the scoring system, four categories rate the potential outcome-specific certainty of evidence: ‘high’, ‘moderate’, ‘low’ and ‘very low’. The NutriGrade scoring tool was modified for the assessment of SRs without MA [8] (Supplementary Material S5). We adjusted the items related to MA: (i) precision: the confidence intervals were deleted, (ii) heterogeneity: this item was reduced to the question about consistency of the results, (iii) publication bias: this item was deleted, (iv) effect size: the RR/HR were deleted and (v) dose–response: this item was deleted. For SRs reporting more than one relevant outcome, each outcome was assessed separately.

### Definition of the outcomes

Throughout the SRs included in this umbrella review and the underlying primary studies, the definitions of outcomes were frequently not consistent. The following outcome specifications were generally used.

Albumin excretion was either reported as albumin excretion per 24 h (24 h) or per minute, or as albumin excretion/liter quantified in 24-h urine samples, or as albumin/creatinine ratio in spot samples. Some studies used the classification microalbuminuria (30 mg–300 mg per 24 h or per mg creatinine) as an outcome, where excretion levels > 300 mg represent macroalbuminuria.

GFR, in addition to albumin excretion, also belongs to the most important indicators regularly determined to assess kidney function. Of those primary studies examining GFR, only a few used direct measurement methods to determine this outcome. Most studies applied one of several published estimating equations either based on a serum or a plasma concentration measurement of creatinine or cystatin C. Equations estimating GFR (eGFR), most frequently used, were Chronic Kidney Disease Epidemiology Collaboration (CKD-EPI), Modification of Diet in Renal Disease (MDRD), or Cockroft Gault (CG). Measured or estimated GFR (eGFR) was in general reported as “mL/min per 1.73 m^2^”. A GFR below 60 ml/min/1.73 m^2^ usually marks a relevant, still mild to moderate (if not lower than 45 ml/min/1.73 m^2^), decline in kidney function [11] and this cutoff (< 60 mL/min/1.73 m^2^) was used in respective primary studies to exclude subjects with an insufficient kidney health. Some studies simply reported serum creatinine concentrations without applying one of the GFR estimation formulas.

CKD, when used as an outcome, was assessed as incident CKD applying the eGFR stage < 60 mL/min per 1.73 m^2^ as criterion.

Kidney stones, used as the assessment outcome in two SRs, were mostly based on self-reported diagnoses. In general, calcium oxalate is the most common stone type, followed by carbonate apatite, uric acid, struvite, brushite and cystine [12].

Urinary calcium excretion, if elevated, is a major and common risk factor for both calcium oxalate and calcium phosphate stone formation [13, 14]. Urinary calcium excretion was measured in 24-h urine samples and reported as excretion rate per 24 h.

Urinary pH is an independent risk factor for the formation of various types of kidney stones and is regarded as an indicator of renal function. Urinary pH is usually determined in 24-h urines, applying pH meters.

Urea and uric acid blood concentrations are protein intake-related markers of renal elimination function for water-soluble, nitrogen-containing metabolic end products. These outcomes were analysed in serum samples, using standard clinical assays, in several primary studies.

### Rating the overall certainty of the evidence

The overall certainty of the evidence was assessed according to the framework outlined in the methodological protocol [8] and in Table 1. For this publication, two authors (TR, RS) made suggestions for rating the overall certainty of evidence. This rating was double-checked by a staff member of the German Nutrition Society (NK) and thereafter reviewed by all co-authors. The final ratings of the overall certainty of evidence was approved by all authors. In an amendment step, rating of the overall certainty of evidence was complemented by two authors (TR and RS) with a specific evaluation on whether the outcome may unequivocally and without bias mirror a health-relevant kidney function change and not only a normal physiological adaptation to an altered protein load. The presence of unambiguous pathometabolic consequences of HPI for kidney health was highly doubted if the changes of the respective outcome either (i) reflect physiological adaptation to a higher protein intake level or (ii) (may) represent physiological consequences of literature-known, but not-considered factors co-varying with the protein amount ingested.

**Table 1:** Grading the overall certainty of evidence according to methodological quality, outcome-specific certainty of evidence, biological plausibility and consistency of results, and definition of the overall certainty of evidence in a modified form according to the GRADE approach [a,b]

Overall certainty of evidence	Underlying criteria	Definition/explanation
Convincing	At least one SR with or without MA of prospective studies available	There is high level of confidence that the true effect lies close to that of the estimate(s) of the effect
	If more than one SR with or without MA are available: all overall results must be consistent^1^	
	In case of a positive or negative association, biological plausibility is given	
	All included SRs with or without MA must reach at least a “moderate” outcome-specific certainty of evidence^2^; in addition, all included SRs must reach at least a methodological quality^3^ of “moderate”	
Probable	At least one SR with or without MA of prospective studies available	There is moderate confidence in the effect estimate(s): the true effect is likely to be close to the estimate of the effect, but there is a possibility that it is substantially different
	If more than one SR with or without MA are available, the majority of overall results must be consistent^1^	
	In case of a positive or negative association, biological plausibility is given	
	The majority^4^ of included SRs with or without MA must have reached at least a “moderate” certainty of evidence^2^; in addition, all included SRs must reach at least a methodological quality^3^ of “moderate”	
Possible	At least one SR with or without MA of prospective studies available	Confidence in the effect estimate(s) is limited: the true effect may be substantially different from the estimate of the effect
	If more than one SR with or without MA are available, the majority of overall results must be consistent^1^	
	In case of a positive or negative association, biological plausibility is given	
	The majority^4^ of included SRs with or without MA must reach at least a “low” certainty of evidence^2^; in addition, the majority^4^ of all included SRs must reach at least a methodological quality^3^ of “moderate”	
Insufficient	No SR is available	There is very little confidence in the effect estimate (s): the true effect is likely to be substantially different from the estimate of effect
	*OR*	
	The majority^4^ of included SRs with or without MA reach a “very low” certainty of evidence^2^; in addition, the majority of all included SRs reach a methodological quality^3^ of “low”	

*MA* meta-analysis, *SR* systematic review

^1^Consistent = overall results of the SR have to be consistently either risk reducing or risk elevating or consistently showing no risk association

^2^Outcome-specific certainty of evidence refers to the NutriGrade rating

^3^Methodological quality refers the AMSTAR 2 rating; SRs graded as “critically low” by AMSTAR 2 are not considered

^4^Majority: > 50% of the included SRs

^a^Alonso-Coello P, Schünemann HJ, Moberg J et al. (2016) GRADE Evidence to Decision (EtD) frameworks: a systematic and transparent approach to making well informed healthcare choices. 1: Introduction. BMJ 353:i2016. https://doi.org/10.1136/bmj.i2016

^b^Kroke A, Schmidt A, Amini AM, Kalotai N, Lehmann A, Bauer JM, Bischoff-Ferrari HA, Boeing H, Egert S, Ellinger S, Kühn T, Louis S, Lorkowski S, Nimptsch K, Remer T, Schulze MB, Siener R, Stangl GI, Volkert D, Zittermann A, Buykens AE, Watzl B, Schwingshackl L (2022) Dietary protein intake and health-related outcomes: a methodological approach for the evidence-based guideline of the German Nutrition Society. Eur J Nutr 10.1007/s00394-021–02,789-5

## Results

**Fig. 1:**
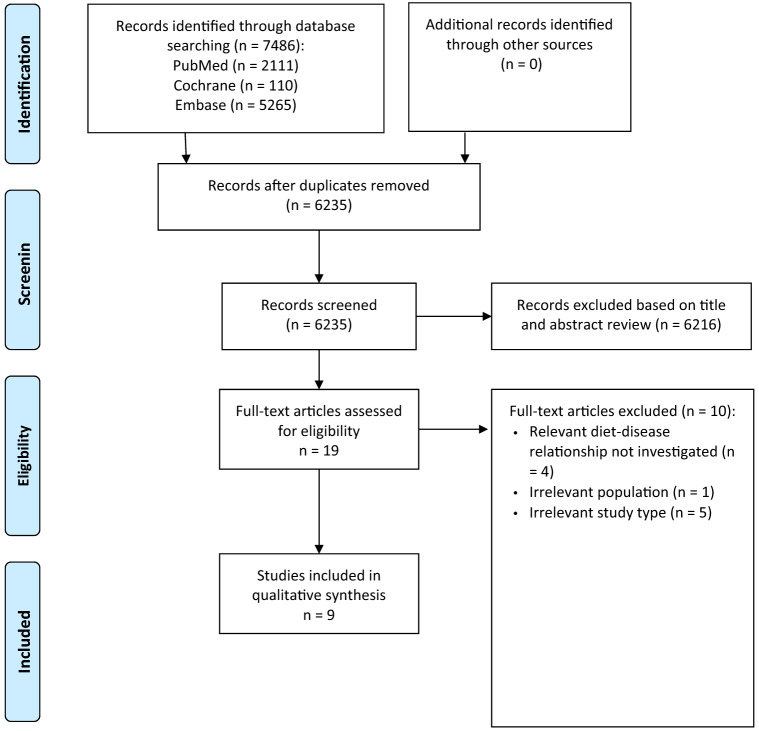
Flow diagram on systematic reviews included

Of the 7486 publications initially identified, 9 SRs remained for analysis: 6 SRs with MA and 3 SRs without MA [15–23]. The study selection process is outlined in Fig. 1. A list of the excluded SRs after full-text screening, including justifications for exclusion, is provided in Supplementary Material S6. None of the SRs was excluded due to ‘critically low’ rating by AMSTAR 2.

### Characteristics of the included systematic reviews

Table 2 shows the characteristics of the SRs. Three of them were SRs with MA of RCTs [16, 18, 19], three were SRs with MA of cohort studies [20–22] and three were SRs without MA of RCTs or cohort studies [15, 17, 23]. One SR with MA conducted a dose–response analysis [16].

**Table 2:** Characteristics of the included systematic reviews

Author, year	Study type, study period	Study population	Exposition	Protein intake	Outcome	Effect estimates	Heterogeneity estimators	NutriGrade rating	AMSTAR 2 rating
Asoudeh 2022 [22]	SR with MA of cohort studies Published until July 2021 Study duration: 6–26 yrs	Both sexes General population Aged ≥ 20 yrs		NP	Risk of kidney stones	Pooled RR (95% CI), random/fixed effect model			Moderate
	2 cohort studies	*n* = 154,221 *n* = 2,982 cases	Total protein			Fixed effect model: 1.04 (0.92, 1.18) Dose-response analysis: 0.99 (0.98, 1.01) per 10g/d increase P_nonlinearity_ = 0.11	I^2^ = 68% *P* = 0.08 I^2^ = 44.7% *P* = 0.18	Low: 4.0	
	6 cohort studies	*n* = 466,109 *n* = 8,494 cases	Animal protein			Random effect model: 1.00 (0.89–1.14)	I^2^ = 62% *P* = 0.02	Low: 4.9	
	5 cohort studies	*n* = 387,816 *n* = 6,542 cases				Dose–response analysis: 0.99 (0.97, 1.02) per 10 g/d increase P_nonlinearity_ = 0.26	I^2^ = 72% *P* < 0.01		
	2 cohort studies	*n* = 320,896 *n* = 8,971 cases	Nondairy animal protein			Fixed effect model: 1.11 (1.03, 1.20) Dose–response analysis: 1.01 (1.00, 1.02) per 10 g/d increase P_nonlinearity_ = 0.38	I^2^ = 0% *P* = 0.47 I^2^ = 42% *P* = 0.16	Low: 5.0	
	2 cohort studies	*n* = 320,896 *n* = 8,971 cases	Dairy protein			Fixed effect model: 0.91 (0.84, 0.99) Dose-response analysis: 0.96 (0.93, 0.99) per 10g/d increase P_nonlinearity_ = 0.86	I^2^ = 0% *P* = 0.55 I^2^ = 0% *P* = 0.57	Moderate. 6.0	
Hengeveld 2022 [23]	SR without MA of RCTs Published before 04/2020 Study duration: 12 wks to 18 mos	Both sexes Relatively healthy and/or people with (moderate) physical function limitations, overweight, obesity and/or (pre-)frailty Aged ≥ 65 yrs	High *vs* low animal protein intake (2 RCTs with concomitant exercise in control and intervention group)	1.06–1.4 g/kg BW/d vs. 0.81-1.05 g/kg BW/d			NA		Moderate
	4 RCTs	*n* = 347–361			Serum creatinine	3 out of 4 RCTs reported no significant effect of increased protein		Low: 4.5	
	5 RCTs	*n* = 622			eGFR	None of the RCTs reported a significant effect of increased protein		Moderate: 5.5	
	1 RCT	*n* = 111			Albumin/creatinine ratio	The RCT reported no significant effect of increased protein		Low: 4.5	
Kelly 2021 [21]	SR with MA of cohort studies Published until June 2019 Follow-up: 5.5–21 yrs	Both sexes General population, partly people with T2D, hypertension, dyslipidaemia and/or CVD Aged 30–67 yrs	Higher vs. lower protein intake	NP		OR (95% CI), random effects model			High
	3 cohort studies	*n* = 19,835			Incident CKD	1.08 (0.91, 1.28) *P* = 0.36	I^2^ = 47% Chi^2^ = 3.75 tau^2^ = 0.01 df = 2 *P* = 0.15	Very low: 3.0	
	5 cohort studies	*n* = 18,507			GFR decline	1.07 (0.96, 1.19) *P* = 0.25	I^2^ = 42% Chi^2^ = 12.16 tau^2^ = 0.01 df = 7 *P* = 0.10	Very low: 3.5	
Lin 2020 [20]	SR with MA of 4 cohort studies Published until May 2019 follow-up: 20–26 yrs	Both sexes Aged 25–79 yrs *n* = 271,969	High vs. low animal protein intake	NP	Nephrolithiasis (incident stones)	RR (95% CI), random effects model 1.1 (1.02–1.19) *P* = NP	No heterogeneity	Low: 4.5	High
Devries 2018 [16]	SR with MA of RCTs Published from 1975 until 2016 Intervention duration: 4 d–104 wks	Both sexes Healthy and/or people with obesity and/or hypertension Aged ≥ 18 yrs	High vs. normal/low protein intake	1.2–3.3 g/kg BW/d vs. 0.3–2.6 g/kg BW/d 20–40 En% vs. 12–24 En% 123–150 g/d vs. 46–75 g/d		SMD (95% CI), random effects model			Moderate
	28 RCTs	*n* = 1409			GFR	0.19 (0.07, 0.31) *P* = 0.002 Dose–response analysis (linear) protein intake (g/kg BW/d) *r* = 0.332, *P* = 0.03	I^2^ = 0% Chi^2^ = 15.77 tau^2^ = 0.00 df = 29 *P* = 0.98	Moderate: 7.05	
	14 RCTs	*n* = 1307			ΔGFR	0.11 ( – 0.05, 0.27) *P* = 0.16 Dose–response analysis (linear) protein intake (g/kg BW/d) r = 0.184, *P* = 0.33	I^2^ = 44% Chi^2^ = 30.26 tau^2^ = 0.05 df = 17 *P* = 0.02	Moderate: 6.35	
Van Elswyk 2018 [15]	SR without MA of RCTs and cohort studies Published before 08/2017 Intervention duration: 4 d–8 wks Follow-up: 15–21 yrs	Both sexes Healthy and/or people with metabolic risk factors Aged ≥ 18 yrs	Protein intake consistent with the US RDA (≥ 0.8 g/kg BW/d or 10–15 En%) or higher protein intake (20–35 En% or 10% higher than comparison intake)	1.8–2.5 g/kg BW/d vs. 0.7–1.5 g/kg BW/d			NA		Low
	1 cohort study	*n* = 3798	Total protein, animal protein, plant protein		eGFR	*“Intake of total, plant, and animal protein are not associated with changes in eGFR over time.”*		Low: 4.0	
	13 RCTs	*n* = 235	Total protein, animal protein, plant protein		GFR	8 of 11 RCTs reported Significantly higher GFR in response to increased protein intake		Very low: 2.0	
	4 RCTs	*n* = 60	Total protein, animal protein, plant protein		Uric acid	3 of 4 RCTs reported elevated uric acid in response to increased protein intake		Very low: 3.0	
Schwingshackl 2014 [18]	SR with MA of RCTs Published until 02/2014 Intervention duration: 1 wk–24 mos	Both sexes Generally healthy and/or people with overweight, obesity and/or T2D - Mean age: 22.3–67 yrs	High vs. normal/low protein diet (≥ 5% difference in total energy intake)	12.5–40 En% vs*.* 5.4–24 En% or 1.0–2.4 g/kg BW/d vs*.* 0.5–1.2 g/kg BW/d		Pooled WMD (95% CI), random effects model			Moderate
	21 RCTs	*n* = 1599			GFR	7.18 ml/min/1.73 m^2^ (4.45, 9.91) *P* = < 0.001	I^2^ = 52% tau^2^ = 16.53 Chi^2^ = 47.76 df = 23 *P* = 0.002	Moderate: 6.6	
	22 RCTs	*n* = 1764			Serum creatinine	– 1.42 µmol/l ( – 3.50, 0.65) *P* = 0.18	I^2^ = 57% tau^2^ = 10.13 Chi^2^ = 51.42 df = 22 *P* = 0.0004	Low: 5.6	
	13 RCTs	*n* = 910			Serum urea	1.75 mmol/l (1.13, 2.37) *P* = < 0.00001	I^2^ = 88% tau^2^ = 1.13 Chi^2^ = 112.57 df = 13 *P* < 0.00001	Moderate: 6.6	
	8 RCTs	*n* = 295			Serum uric acid	0.18 µmol/l ( – 0.08, 0.44) *P* = 0.17	I^2^ = 3% tau^2^ = 0.00 Chi^2^ = 7.25 df = 7 *P* = 0.40	Low: 5.4	
	7 RCTs	*n* = 210			Urinary pH	– 0.39 ( – 0.82, 0.03) *P* = 0.07	I^2^ = 95% tau^2^ = 0.33 Chi^2^ = 148.51 df = 7 *P* < 0.00001	Low: 5.3	
	11 RCTs	*n* = 783			Urinary albumin/protein	0.50 mg/24 h ( – 2.83, 3.82) *P* = 0.77	I^2^ = 63% tau^2^ = 13.51 Chi^2^ = 32.58 df = 12 *P* = 0.001	Low: 5.8	
	10 RCTs	*n* = 708			Urinary calcium excretion	25.43 mg/24 h (13.62, 37.24) *P* < 0.001	I^2^ = 90% tau^2^ = 172.58 Chi^2^ = 100.09 df = 10 *P* < 0.00001	Moderate: 6.6	
Pedersen 2013 [17]	SR without MA of RCTs and cohort studies Published between 01/2000 and 12/2011 Intervention duration: 7d–3 wks follow-up: 7–10 yrs	Both sexes Generally healthy and/or people with overweight or obesity Mean age: 24–70 yrs	RCTs: high vs. normal/low protein intake Cohort studies: En% from protein or total protein intake (in kcal or servings)	1–1.5 g/kg BW/d vs. 2–3 g/kg BW/d			NA		Moderate
	2 RCTs	*n* = 48	Total protein, animal protein		GFR	*"The evidence is assessed as inconclusive regarding the relation of protein intake to renal function based on GFR.”*		low: 5.0	
	2 cohort studies	*n* = 10,216	Total protein		eGFR			Low: 3.0	
	2 RCTs	*n* = 48	Total protein		Microalbuminuria	*"The evidence is assessed as inconclusive regarding the relation of protein intake to renal function based on microalbuminuria.”*		Low: 4.5	
			Animal protein					Low: 5.0	
	2 cohort studies	*n* = 10,216	Total protein					Low: 3.0	
	2 cohort studies	*n* = 141,864	Animal protein		Kidney stones	*"The evidence is assessed as inconclusive regarding the relation of protein intake to risk of kidney stones”*		Low: 3.0	
Santesso 2012 [19]	SR with MA of 2 RCTs Published before 08/2011 Intervention duration: 84 d	Both sexes People with overweight or obesity Mean age: 46–58 yrs *n* = 67	High vs. low protein (≥ 5% difference in total energy intake)	Median: 27 En% vs. 18 En% Range: 16–45 En% vs. 5–23 En%	Serum creatinine	Pooled SMD (95% CI), random effects model 6.14 (2.49, 9.79) *P* = 0.001	I^2^ = 0% tau^2^ = 0.00 Chi^2^ = 0.10 df = 2 *P* = 0.95	Very low: 2.0	High

AMSTAR 2, A Measurement Tool to Assess Systematic Reviews; BW, body weight; CI, confidence interval; d, day(s); eGFR, estimated glomerular filtration rate; En%, energy percentage; GFR, glomerular filtration rate; MA, meta-analysis; MD, mean difference; mo, month; NA, not applicable; NP, not provided; OR, odds ratio; RCT, randomised controlled trial; RR, relative risk; SMD, standardised mean difference; SR, systematic review; T2D, type 2 diabetes mellitus; wk, week; WMD, weighted mean difference; yr, year

The SRs investigated kidney stone formation [17, 20], CKD [21], and kidney function-related parameters, i.e. GFR [15–18, 21, 23], urinary albumin excretion [15, 18, 23], urinary calcium excretion [18], urinary pH [15, 18], serum urea [18], serum uric acid [15, 18], and simply serum creatinine, not used for GFR calculation [19, 23].

Seven SRs with or without MA of RCTs or cohort studies investigated the effect or association of total protein intake on or with kidney diseases and selected kidney function-related parameters [15–19, 21, 23]. Two SRs with MA examined the effect of animal protein intake on incident kidney stone formation and one SR without MA investigated the association of animal protein intake on selected kidney function-related parameters [20, 22]. One SR also examined potential differences between animal protein intake and plant protein intake [15]. The intervention duration of the primary studies was 4 days to 24 months for RCTs and 6 to 26 years for cohort studies. Participants were generally healthy, male or female, and aged ≥ 18 years. Some of the primary studies also included participants with metabolic risk factors (overweight, obesity, hypertension, hypercholesterolaemia, or diabetes mellitus type 2) [15–19, 21] or with (moderate) physical function limitations [23].

### Type and range of protein intake

The protein intake ranged from 1.0 to 3.3 g/kg BW/d (high-protein groups) vs*.* 0.3 to 2.6 g/kg BW/d (control groups) or from 12.5 to 40 energy percentage (En%) (high-protein groups) vs*.* 5.4 to 24 En% (control groups) or from 123 to 150 g/d (high-protein groups) vs*.* 46 to 75 g/d (control groups) for all included RCTs investigating the association between protein intake and the outcomes.

No information was provided for the protein intake of included cohort studies in the four SRs [17, 20–22]. One primary study in the SR from Devries et al. reported only ‘unlimited protein consumption’ [16].

### Methodological quality and outcome-specific certainty of the evidence

For each included SR, overall scores of AMSTAR 2 and NutriGrade are summarised in Table 2. Supplementary Materials S7 and S8 provide a more detailed description showing the assessments of each individual item. Methodological quality as assessed with AMSTAR 2 was rated ‘high’ for three SRs [19–21], moderate for five SRs [16–18, 22, 23], and ‘low’ for one SR [15].

The outcome-specific certainty of evidence as assessed with NutriGrade was moderate for increases in GFR (standardised mean difference (SMD): 0.19; 95% CI 0.07, 0.31; I^2^ = 0% [16], mean difference (MD): 7.18 ml/min/1.73 m^2^; 95% CI 4.45, 9.91; I^2^ = 52%) [18], urinary calcium excretion (MD: 25.43 mg/24 h; 95% CI 13.62, 37.24; I^2^ = 90%) [18], and serum urea (MD: 1.75 mmol/l; 95% CI 1.13, 2.37; I^2^ = 88%) [18]; ‘low’ for constancy of urinary albumin [18], serum uric acid [18], and urinary pH [18]; and also ‘low’ in two other SRs for GFR [17] and serum creatinine constancies [23]. A ‘very low’ outcome-specific certainty of evidence was additionally seen for constancy of GFR [21], as well as for increases in GFR (8 out of 13 RCTs were erroneously reported by van Elswyk et al. [15] instead of 8 out of 11 RCTs that actually showed significantly higher GFR in response to increased protein intake (see Discussion)) [15], and increases in serum uric acid (3 of 4 RCTs reported elevated serum uric acid in response to increased protein intake) [15] and serum creatinine (SMD: 6.14; 95% CI 2.49, 9.79; I^2^ = 0%) [19]. In the latter SR of Santesso et al. [19], only two out of six identified primary studies on serum creatinine were taken into account. None of these kidney function-related outcomes except the urine pH showed any substantial inverse relationship with the amounts of protein ingested. Based on one SR with MA of cohort studies, a NutriGrade rating of ‘low’ was obtained for a 10% increase (RR: 1.1; 95% CI, 1.02–1.19) in the risk of incident stone formation with the exposition variable animal protein intake [20], while two other SRs (one without and one with MA) found the risk inconclusive [17] or found no effect of animal protein on the risk of incident stone formation [22].

### Rating of the overall certainty of the evidence

Using the criteria outlined in Table 1, the overall certainty of evidence was rated as ‘possible’ for albuminuria to be not elevated and ‘possible’ for urinary pH to be not reduced by HPI. It was also rated as ‘possible’ for GFR, and as ‘probable’ for urinary calcium excretion and serum urea each to be physiologically (regulatorily) and not per se pathophysiologically elevated (for further details see “Discussion”). The rating of the overall certainty of evidence was ‘insufficient’ for the relationship between protein intake and serum uric acid, but it was ‘possible’ for the absence of an association between the exposition variable animal protein intake and the risk of incident kidney stone formation.

## Discussion

This umbrella review, including 6 SRs with MA and 3 SRs without MA, examined the implications of HPI for kidney health. Key findings are that for daily protein ingestion above dietary recommendations, no convincing evidence could be ascertained for kidney function decline relevant relationships with urinary albumin excretion, renal GFR, and kidney stone risk. Also for the further assessed renal-related outcomes, none of the gradings of the overall certainty of evidence led to an assessment as ‘possible’ or ‘probable’ for detrimental HPI influences on kidney function.

According to the criteria given in Table 1, for the risk marker of CKD albumin excretion [11], the overall certainty of evidence was graded as ‘possible’ to be not elevated through HPI in both young and elderly healthy adults. Furthermore, overall certainty of evidence was graded as ‘possible’ for GFR and ‘probable’ for urinary calcium excretion, as well as for serum urea, to be physiologically (regulatorily) increased with HPI. It is noteworthy that this grading as ‘possible’ for GFR was partly due to a downgrading effect through the only SR [15] with an overall low methodological quality (AMSTAR 2) and a substantial miscategorisation of the outcome GFR [15]. Instead of 8 out of 13 RCTs with GFR determinations (< 2/3), as reported by the authors, actually 8 out of 11 RCTs with GFR determinations (> 2/3) showed higher GFRs with HPI [15], thus rather allowing an assessment of the overall certainty of evidence as ‘almost probable’ and not just ‘possible’.

As outlined below, the elevations of most of the outcomes along with HPI have to be interpreted cautiously, i.e. mostly as physiological regulatory responses and not as pathophysiological increases. However, it should be considered that an elevated urinary calcium excretion may represent a risk factor for calcium stone formation. Nevertheless, for kidney stone disease, ‘possible’ evidence was derived for an absence of an association with higher animal protein intake [17, 20].

As HPI has been associated with metabolic changes that can exhibit a risk for kidney stone formation in healthy individuals [4, 24], this issue is addressed in the following along with further specific comments on the examined outcomes. Physiological background explanations are provided for a far-reaching inappropriateness of urinary calcium excretion, urinary pH, serum urea, serum uric acid, and even of the important kidney function parameter GFR (or serum creatinine) as unbiased renal health outcomes for examinations in (mostly) healthy populations if no specific adjustments are conducted. Accordingly, the suitability of these parameters to unbiasedly reveal pathophysiologically relevant influences of HPI on kidney health will be critically appraised.

### Albumin excretion

The current NutriGrade ratings of ‘low’ for the finding that the diagnostically important kidney parameter urinary albumin excretion and dietary protein intake are unrelated, definitely prompting that this potential absence of an albuminuria-elevating effect through HPI needs to be further examined and particularly studied for observation periods longer than 2 years.

### Kidney stones

The prevalence of urinary stone disease in the general population has been reported to range between 4.7% and 8.8% [25, 26]. Kidney stone formation is associated with an elevated risk of chronic and end-stage kidney disease, probably due to kidney injury from obstructive nephropathy [27, 28]. An HPI may promote the risk of stone formation by providing an acid load that could lead to several metabolic changes, including decreases in urinary pH and citrate excretion, and increases in urinary calcium and uric acid excretion [24, 29–31]. A higher dietary net acid load, estimated by animal protein-to-potassium ratio or net acid excretion (NAE), was associated with a higher risk of kidney stone formation in large observational studies [32]. These data suggest that the proportion of the consumed amount of alkalising fruits and vegetables compared to the total amount of ingested protein could modify the risk of HPI for kidney stone formation. It is beyond controversy that fruits and vegetables have a marked alkalising potential and can in such a way relevantly neutralise the proton load, metabolically generated from ingested protein [33, 34]. High dietary acidity, resulting in lower urine pH, is a risk factor for several kidney stone types, particularly for the most common, i.e. calcium oxalate stones. The higher the urine pH, the higher is the stone-inhibiting citrate excretion and calcium-binding capacity and the lower is the urinary calcium excretion [35].

In conclusion, a number of protein intake-related, metabolic, and idiopathic risk factors and confounders, such as low or high urine pH, hypercalciuria, hypocitraturia, hyperuricosuria, hyperoxaluria and further dietary/environmental risk factors, such as high sodium chloride intake and low urine volume [36, 37], all complicate a straight examination of ‘the inherent impact of protein’ on stone formation. However, most of these risk factors can at least partly be avoided or reduced by the respective changes in dietary habits, e.g. by increasing the habitual intake of metabolically alkalising fruits and vegetables [32].

### GFR

Increases in GFR frequently occur during the first years after onset of diabetes mellitus type 1 or 2 [38]. This phenomenon is termed glomerular hyperfiltration. With advancing duration of the disorder, hyperfiltration regresses again and frequently turns into a pathophysiological decline of GFR. Increased body fatness and obesity also lead to elevations in GFR, independent of hyperglycaemia and other metabolic and hormonal signals also present in diabetes.

Another major stimulus of GFR is protein intake. GFR increases, lasting for several hours, occur after protein-rich meals [39], implying that if HPI and blood sampling are temporally relatively far apart (overnight fasting or even longer), GFR increases can, but may not necessarily, be any longer detectable with the use of mere serum measurement-based estimates (eGFR), although they would be observable by 24-h urine-based GFR measures. This is one of a number of explanations why in all MAs and SRs that included the outcome GFR, at least some primary studies were present which did not find GFR increases following increased protein ingestion by healthy subjects. In principle, elevations in GFR are basic, physiologically adaptive mechanisms induced by HPI in case of normal kidney function state [39–43].

In line herewith, none of the SRs of this umbrella review found clear indications for a GFR reduction due to HPI. Accordingly, one could classify GFR increases or at least GFR stability as a very probable consequence of raises in protein intake above dietary recommendations in the healthy state, despite the fact that the formal use of the modified grading system [8] only resulted in a grading of ‘possible’ for the overall certainty of evidence for the absence of GFR reductions.

Among others, not only younger age (until around 35 years) [44] and HPI [39–43], but also increases in BW [41], BMI or fat mass [45, 46], insulin resistance [47, 48], insulin secretion [49], and sodium chloride intake [45, 50, 51] all have a GFR-elevating potential. Accordingly, the examination of a potential kidney function decline by using GFR reduction as a marker or an outcome in initially metabolically healthy subjects appears – at first glance – not ideal for an exposure that by itself biologically raises GFR. GFR changes, however, should be studied in the future (as far as possible bias free) as a major outcome for the assessment of gradual kidney function decline over periods of more than 5–10 years by more appropriately controlling relevant confounders.

### Urinary calcium excretion

Various dietary factors affect urinary calcium excretion, particularly the intakes of calcium, protein, and sodium chloride. In healthy subjects, intestinal calcium absorption is approximately 25% [52]. However, intestinal hyperabsorption of calcium is frequently diagnosed in stone formers [53]. Higher dietary protein intakes are consistently reported to increase urinary calcium excretion [18, 54, 55], in part due to the increased GFR [56] (see above). Apart from calcium and protein intake, urinary calcium excretion is also related to urinary NAE and acidotic stimuli [57]. While the administration of 1.5 g/d L-methionine did not significantly raise urinary calcium excretion in healthy subjects [58], the supplementation of 3 g/d L-methionine resulted in a significant increase in urinary calcium excretion by about 1 mmol/d (40 mg/d) in parallel with a rise in urinary NAE of 40 mEq/d [59]. Accordingly, without specific adjustments for the aforementioned confounding influences, the utilisation of urinary calcium excretion as an important urolithiasis-related renal health outcome appears to be less useful.

### Urinary pH

Urinary pH marks the small amount of free hydrogen ions (H^+^) not buffered by ammonia and titratable acid (i.e. mostly phosphate) and reflects, to some degree, the overall excess of H^+^ that is renally secreted. The overall, i.e. the buffered amount of H^+^ daily eliminated by the kidney is quantified as NAE [60–62]. Although 24-h urine pH and NAE/d usually show good correlations [34, 63], a variation in renal buffer supply can markedly change the usual pH–NAE relationship. One major confounder in this regard is protein intake itself. The higher the protein intake, the higher is the kidney´s capacity to excrete surplus H^+^ [64, 65]. Accordingly, if protein intake increases and NAE is constant (through higher alkali intake), the ammonia buffer is much more easily renally provided. This means that a lower free proton stress (a lower H^+^ signalling) is required to increase buffer provision, i.e. ammoniagenesis. Correspondingly, in subjects without kidney disease, urine pH will be higher with HPI for every given acid load, i.e. for a constant potential renal acid load (PRAL) or a constant NAE. Thus, 24-h urine pH can only be used as a marker for kidney function change if measurements of renal 24-h NAE or PRAL are concurrently performed and appropriately adjusted for [66]. Even with HPI of around 80 to 100 g/d, mean 24-h urine pH can be kept at ≥ 6 through moderate alkali equivalent ingestion [33, 64]. Besides this, HPI with a higher NAE, higher age (> 50 years) [67], higher BMI or body fat [66, 68, 69], and other features of the metabolic syndrome including insulin resistance [68, 70] each contribute to urine pH reductions. Thus, the NutriGrade rating of ‘low’ obtained for a potential constancy of the urine pH along with rises in protein intake [18] suggests that HPI does not necessarily increase renal “free proton stress”. However, the examination of urinary free protons at least in combination with (reliable markers of) protein intake and the related net acid load can be a valuable tool to assess renal acid excretion function as well as stone formation risk [66, 71].

### Serum urea and serum uric acid

Increases in urea, mostly within the normal physiological range, have been reported in almost all primary studies after protein intake was raised. Since elevation of serum urea above the upper limit of the normal range primarily depends on functional GFR reduction [72] and further confounders like hydration status, circulating urea rather represents an insensitive indicator of kidney function [72]. Next, serum uric acid shows varying interdependences with protein ingestion [73], purine intake, hydration status [74], and GFR [75], as well as with metabolic syndrome [76] and diabetes mellitus type 2 [77]. Thus, irrespective of the rating of the overall certainty of evidence as ‘probable’ or ‘insufficient’ for effects due to increases in protein intake, the utilisation of serum urea and uric acid, respectively, for the assessment of potential influences of HPI on kidney health appears to be a less specific approach.

### Strengths and limitations

A strength of this umbrella review is that six of the included hitherto published SRs on the relevance of HPI for kidney health comprise, either exclusively or primarily, RCTs. A further strength is that we critically examined in more detail the suitability, as well as relevant physiological confounders, of those kidney parameters commonly used to investigate kidney health. However, several limitations have to be taken into account when assessing the findings of the SRs included in this umbrella review. We applied NutriGrade instead of the GRADE approach (Grading of Recommendations, Assessment, Development and Evaluation) because an important novelty of NutriGrade (published in 2016) was the modified classification for MA of RCTs and cohort studies compared with the traditional GRADE approach (initially classifying RCTs with an initial high score and cohort studies with a low score) [78]. We are aware that in the meantime, the GRADE approach was amended (adjustments published in 2019, but after the guideline methodology was established in 2017) in a way that cohort studies can now also be assigned an initially high score, when risk of bias tools such as ROBINS-I are used [79]. The intervention duration of the primary RCTs and also the protein intake levels varied considerably with ranges from 1 week to 2 years, and intakes from 12.5 to 40 En% solely in the high-protein groups, respectively. Although dietary protein sources have been provided in most primary studies, more specific statements regarding the relevance of animal vs. plant vs. dairy protein could not be drawn, particularly due to an insufficient number of corresponding specific data analyses. Furthermore, the substantial degree of heterogeneity, present for the different outcomes, could not be further assessed. Further important limitations of the current umbrella review are that (i) major primary studies, not included in the SRs or MAs, remained unconsidered and (ii) that, of the nine SRs that could be included, only 3 examined the most diagnostically conclusive outcome variable, i.e. albuminuria as well as kidney stones. The various other kidney function-related outcomes that were examined, however, showed clear weaknesses regarding a specific, i.e. an unconfounded assessment of possible kidney function impairments. Their increases (or potential urinary pH reductions) along with HPI are biologically plausible, but without direct specific pathophysiological relevance.

## Conclusion

For none of the outcomes was a ‘convincing’ certainty of evidence found for detrimental effects of HPI with regard to the development of kidney diseases. However, most of the included studies were of rather short-term duration, so that a possible long-term risk over decades cannot be assessed at present. Although the overall certainty of evidence has been rated as ‘probable’ for an increase in urinary calcium excretion, a risk factor for calcium stone formation, the rating of the overall certainty of evidence revealed no relationship between protein intake and the risk of incident nephrolithiasis. Detailed future research is required into whether albumin excretion actually does not increase and GFR does not fall through protein intake levels exceeding the dietary recommendation of 0.8 g/kg BW/d over periods of more than 2 years and after decades in older age. Such long-term confirmatory studies, adequately controlled for the specified confounders, are necessary before changing or adapting statements on higher protein intake levels as being “quasi safe” or recommendable.

## Supplementary Information

Below is the link to the electronic supplementary material.

Supplementary file1 (DOCX 19 KB)

Supplementary file2 (XLSX 11 KB)

Supplementary file3 (PDF 405 KB)

Supplementary file4 (PDF 316 KB)

Supplementary file5 (PDF 293 KB)

Supplementary file6 (DOCX 16 KB)

Supplementary file7 (DOCX 21 KB)

Supplementary file8 (XLSX 32 KB)

Supplementary file9 (XLSX 14 KB)

## Data Availability

Not applicable.

## Abbreviations

AMSTAR 2: A measurement tool to assess systematic reviews 2
BW: Body weight
d: Day
En%: Energy percentage
CKD: Chronic kidney disease
GFR: Glomerular filtration rate
HPI: Higher protein intake
MA: Meta-analysis/meta-analyses
MD: Mean difference
NAE: Net acid excretion
RCT: Randomised controlled trial
SMD: Standardised mean difference
SR: Systematic review(s)
