# Supplementary material for: Protein intake and risk of urolithiasis and kidney diseases: an umbrella review of systematic reviews for the evidence-based guideline of the German Nutrition Society
Source: Eur J Nutr. 2023 May 3;62(5):1957–75. doi: 10.1007/s00394-023-03143-7 (PMC10349749; doi:10.1007/s00394-023-03143-7)
Supplement: Supplementary file 1 — Supplementary file1 (DOCX 19 KB) [file 394_2023_3143_MOESM1_ESM.docx]

Supplementary Material S1. Search strategy.

|  | **Database** | | |
| --- | --- | --- | --- |
| **Research**  **topic** | **PubMed** | **Cochrane** | **Embase** |
| **Study types** | Meta-analy* [tiab] OR "meta-analysis" [tiab] OR "meta analyses" [tiab] OR "meta analysis" [tiab] OR metaanalysis [tiab] OR "meta-analyze" [tiab] OR "meta-analysis" [Publication Type] OR systematic [sb]^1^ OR "systematic review" [tiab] | - | 'Meta analysis'/exp OR 'systematic review'/exp OR meta-analy*:ti,ab OR 'meta-analysis':ti,ab OR 'meta analyses':ti,ab OR 'meta analysis':ti,ab OR metaanalysis:ti,ab OR 'meta-analyze':ti,ab OR 'systematic review':ti,ab |
| **Protein** | "dietary proteins" [mh] OR "diet, protein-restricted" [mh] OR "whey proteins" [mh] OR protein [tiab] OR proteins [tiab] OR "high-protein" [tiab] OR "low-protein" [tiab] OR "whey powder" [tiab] OR "whey powders" [tiab] OR "hypoprotein diet" [tiab] OR "peptidyl group" [tiab] OR "dairy product" [tiab] OR "dairy products" [tiab] OR "protein-free" [tiab] OR "protein-restricted" [tiab] | [mh "dietary proteins"] OR [mh "diet, protein-restricted"] OR [mh "whey proteins"] OR protein:ti,ab OR proteins:ti,ab OR "high-protein":ti,ab OR "low-protein":ti,ab OR "whey powder":ti,ab OR "whey powders":ti,ab OR "hypoprotein diet":ti,ab OR "peptidyl group":ti,ab OR "dairy product":ti,ab OR "dairy products":ti,ab OR "protein-free":ti,ab OR "protein-restricted":ti,ab | 'protein intake'/exp OR 'protein restriction'/exp OR 'dairy product'/exp OR 'yolk protein'/exp OR 'proteins by anatomical concept'/exp OR 'proteins by organism'/exp OR protein:ti,ab OR proteins:ti,ab OR 'high-protein':ti,ab OR 'low-protein':ti,ab OR 'whey powder':ti,ab OR 'whey powders':ti,ab OR 'hypoprotein diet':ti,ab OR 'peptidyl group':ti,ab OR 'dairy product':ti,ab OR 'dairy products':ti,ab OR 'protein-free':ti,ab OR 'protein-restricted':ti,ab |
| **Kidney health** | kidney [mh] OR "kidney function tests" [mh] OR "kidney diseases" [mh] OR "renal circulation" [mh] OR urolithiasis [mh] OR kidney [tiab] OR kidneys [tiab] OR kidney* [tiab] OR nephron [tiab] OR nephrons [tiab] OR nephrotic [tiab] OR nephritic [tiab] OR "split function test" [tiab] OR perinephritis [tiab] OR urolithiasis [tiab] OR urolithiasis* [tiab] OR nephrolithiasis [tiab] OR nephrolithiasis* [tiab] OR lithiasis [tiab] OR lithiasis* [tiab] OR calculi [tiab] OR calculus [tiab] OR "coral stone" [tiab] OR "stone, urinary tract" [tiab] OR "stone, urine" [tiab] OR "urinary stone" [tiab] OR "urinary tract stone" [tiab] OR "urine stone" [tiab] OR urolith [tiab] OR renal [tiab] OR intrarenal [tiab] OR creatinine [tiab] OR microalbuminuria [tiab] OR albumin [tiab] OR albuminuria [tiab] OR ACR [tiab] OR "glomerular filtration rate" [tiab] OR "glomerular filtration rates" [tiab] OR "glomerulus filtration rate" [tiab] OR "glomerulus filtration rates" [tiab] OR "glomerulofiltration rate" [tiab] OR "filtration rate, glomerular" [tiab] OR "filtration rates, glomerular" [tiab] OR "rate, glomerular filtration" [tiab] OR "rates, glomerular filtrations" [tiab] OR GFR [tiab] OR eGFR [tiab] OR hyperfiltration [tiab] OR nephropathy [tiab] OR KD [tiab] OR CKD [tiab] OR ESKD [tiab] OR ESRD [tiab] OR "advanced glycation end product" [tiab] OR "advanced glycation end products" [tiab] OR proteinuria [tiab] OR uremia [tiab] OR hemodialysis [tiab] OR haemodialysis [tiab] OR "peritoneal dialysis" [tiab] OR anuria [tiab] OR "hepatorenal syndrome" [tiab] OR hydronephrosis [tiab] OR pyonephrosis [tiab] OR hyperoxaluria [tiab] OR "renovascular hypertension" [tiab] OR "hypertension, renovascular" [tiab] OR nephritis [tiab] OR glomerulonephritis [tiab] OR pyelitis [tiab] OR nephrocalcinosis [tiab] OR nephrosclerosis [tiab] OR nephrosis [tiab] OR perinephritis [tiab] OR "cardio-renal syndrome" [tiab] OR hypophosphatemia [tiab] OR pseudohypoaldosteronism [tiab] OR azotemia [tiab] OR "hemolytic-uremic syndrome" [tiab] OR "tubulo-interstitial" [tiab] OR acidosis [tiab] OR ammoniagenesis [tiab] OR ammonia [tiab] OR ammonium [tiab] OR hypokalemia [tiab] OR hyperkalemia [tiab] OR "serum bicarbonate" [tiab] OR "blood bicarbonate" [tiab] OR "urine-pH" [tiab] | [mh kidney] OR [mh "kidney function tests"] OR [mh "kidney diseases"] OR [mh "renal circulation"] OR [mh urolithiasis] OR kidney:ti,ab OR kidneys:ti,ab OR kidney*:ti,ab OR nephron:ti,ab OR nephrons:ti,ab OR nephrotic:ti,ab OR nephritic:ti,ab OR "split function test":ti,ab OR perinephritis:ti,ab OR urolithiasis:ti,ab OR urolithiasis*:ti,ab OR nephrolithiasis:ti,ab OR nephrolithiasis*:ti,ab OR lithiasis:ti,ab OR lithiasis*:ti,ab OR calculi:ti,ab OR calculus:ti,ab OR "coral stone":ti,ab OR "stone, urinary tract":ti,ab OR "stone, urine":ti,ab OR "urinary stone":ti,ab OR "urinary tract stone":ti,ab OR "urine stone":ti,ab OR urolith:ti,ab OR renal:ti,ab OR intrarenal:ti,ab OR creatinine:ti,ab OR microalbuminuria:ti,ab OR albumin:ti,ab OR albuminuria:ti,ab OR ACR:ti,ab OR "glomerular filtration rate":ti,ab OR "glomerular filtration rates":ti,ab OR "glomerulus filtration rate":ti,ab OR "glomerulus filtration rates":ti,ab OR "glomerulofiltration rate":ti,ab OR "filtration rate, glomerular":ti,ab OR "filtration rates, glomerular":ti,ab OR "rate, glomerular filtration":ti,ab OR "rates, glomerular filtrations":ti,ab OR GFR:ti,ab OR eGFR:ti,ab OR hyperfiltration:ti,ab OR nephropathy:ti,ab OR KD:ti,ab OR CKD:ti,ab OR ESKD:ti,ab OR ESRD:ti,ab OR "advanced glycation end product":ti,ab OR "advanced glycation end products":ti,ab OR proteinuria:ti,ab OR uremia:ti,ab OR hemodialysis:ti,ab OR haemodialysis:ti,ab OR "peritoneal dialysis":ti,ab OR anuria:ti,ab OR "hepatorenal syndrome":ti,ab OR hydronephrosis:ti,ab OR pyonephrosis:ti,ab OR hyperoxaluria:ti,ab OR "renovascular hypertension":ti,ab OR "hypertension, renovascular":ti,ab OR nephritis:ti,ab OR glomerulonephritis:ti,ab OR pyelitis:ti,ab OR nephrocalcinosis:ti,ab OR nephrosclerosis:ti,ab OR nephrosis:ti,ab OR perinephritis:ti,ab OR "cardio-renal syndrome":ti,ab OR hypophosphatemia:ti,ab OR pseudohypoaldosteronism:ti,ab OR azotemia:ti,ab OR "hemolytic-uremic syndrome":ti,ab OR "tubulo-interstitial":ti,ab OR acidosis:ti,ab OR ammoniagenesis:ti,ab OR ammonia:ti,ab OR ammonium:ti,ab OR hypokalemia:ti,ab OR hyperkalemia:ti,ab OR "serum bicarbonate":ti,ab OR "blood bicarbonate":ti,ab OR "urine-pH":ti,ab | 'kidney'/exp OR 'kidney function test'/exp OR 'kidney disease'/exp OR 'kidney circulation'/exp OR 'urolithiasis'/exp OR 'glomerulus filtration rate'/exp OR kidney:ti,ab OR kidneys:ti,ab OR kidney*:ti,ab OR nephron:ti,ab OR nephrons:ti,ab OR nephrotic:ti,ab OR nephritic:ti,ab OR 'split function test':ti,ab OR perinephritis:ti,ab OR urolithiasis:ti,ab OR urolithiasis*:ti,ab OR nephrolithiasis:ti,ab OR nephrolithiasis*:ti,ab OR lithiasis:ti,ab OR lithiasis*:ti,ab OR calculi:ti,ab OR calculus:ti,ab OR 'coral stone':ti,ab OR 'stone, urinary tract':ti,ab OR 'stone, urine':ti,ab OR 'urinary stone':ti,ab OR 'urinary tract stone':ti,ab OR 'urine stone':ti,ab OR urolith:ti,ab OR renal:ti,ab OR intrarenal:ti,ab OR creatinine:ti,ab OR microalbuminuria:ti,ab OR albumin:ti,ab OR albuminuria:ti,ab OR ACR:ti,ab OR 'glomerular filtration rate':ti,ab OR 'glomerular filtration rates':ti,ab OR 'glomerulus filtration rate':ti,ab OR 'glomerulus filtration rates':ti,ab OR 'glomerulofiltration rate':ti,ab OR 'filtration rate, glomerular':ti,ab OR 'filtration rates, glomerular':ti,ab OR 'rate, glomerular filtration':ti,ab OR 'rates, glomerular filtrations':ti,ab OR GFR:ti,ab OR eGFR:ti,ab OR hyperfiltration:ti,ab OR nephropathy:ti,ab OR KD:ti,ab OR CKD:ti,ab OR ESKD:ti,ab OR ESRD:ti,ab OR 'advanced glycation end product':ti,ab OR 'advanced glycation end product':ti,ab OR proteinuria:ti,ab OR uremia:ti,ab OR hemodialysis:ti,ab OR haemodialysis:ti,ab OR 'peritoneal dialysis':ti,ab OR anuria:ti,ab OR 'hepatorenal syndrome':ti,ab OR hydronephrosis:ti,ab OR pyonephrosis:ti,ab OR hyperoxaluria:ti,ab OR 'renovascular hypertension':ti,ab OR 'hypertension, renovascular':ti,ab OR nephritis:ti,ab OR glomerulonephritis:ti,ab OR pyelitis:ti,ab OR nephrocalcinosis:ti,ab OR nephrosclerosis:ti,ab OR nephrosis:ti,ab OR perinephritis:ti,ab OR 'cardio-renal syndrome':ti,ab OR hypophosphatemia:ti,ab OR pseudohypoaldosteronism:ti,ab OR azotemia:ti,ab OR 'hemolytic-uremic syndrome':ti,ab OR 'tubulo-interstitial':ti,ab OR acidosis:ti,ab OR ammoniagenesis:ti,ab OR ammonia:ti,ab OR ammonium:ti,ab OR hypokalemia:ti,ab OR hyperkalemia:ti,ab OR 'serum bicarbonate':ti,ab OR 'blood bicarbonate':ti,ab OR 'urine-pH':ti,ab |

/exp, exploded Emtree terms; mh, MeSH terms; [sb], subject search; ti,ab/tiab, title/abstract;

^1^ PubMed has changed the search strategy of its [sb]-filter for identifying systematic reviews in 01/2019. To maintain continuity we used this previous version for all our literature searches: (systematic review [ti] OR meta-analysis [pt] OR meta-analysis [ti] OR systematic literature review [ti] OR this systematic review [tw] OR pooling project [tw] OR (systematic review [tiab] AND review [pt]) OR meta synthesis [ti] OR meta synthesis [ti] OR integrative review [tw] OR integrative research review [tw] OR rapid review [tw] OR consensus development conference [pt] OR practice guideline [pt] OR drug class reviews [ti] OR cochrane database syst rev [ta] OR acp journal club [ta] OR health technol assess [ta] OR evid rep technol assess summ [ta] OR jbi database system rev implement rep [ta]) OR (clinical guideline [tw] AND management [tw]) OR ((evidence based[ti] OR evidence-based medicine [mh] OR best practice* [ti] OR evidence synthesis [tiab]) AND (review [pt] OR diseases category[mh] OR behavior and behavior mechanisms [mh] OR therapeutics [mh] OR "evaluation studies" [pt] OR “validation studies” [pt] OR guideline [pt] OR pmcbook)) OR ((systematic [tw] OR systematically [tw] OR critical [tiab] OR (study selection [tw]) OR (predetermined [tw] OR inclusion [tw] AND criteri* [tw]) OR exclusion criteri* [tw] OR main outcome measures [tw] OR standard of care [tw] OR standards of care [tw]) AND (survey [tiab] OR surveys [tiab] OR overview* [tw] OR review [tiab] OR reviews [tiab] OR search* [tw] OR handsearch [tw] OR analysis [ti] OR critique [tiab] OR appraisal [tw] OR (reduction [tw] AND (risk [mh] OR risk [tw]) AND (death OR recurrence))) AND (literature [tiab] OR articles [tiab] OR publications [tiab] OR publication [tiab] OR bibliography [tiab] OR bibliographies [tiab] OR published [tiab] OR pooled data [tw] OR unpublished [tw] OR citation [tw] OR citations [tw] OR database [tiab] OR internet [tiab] OR textbooks [tiab] OR references [tw] OR scales [tw] OR papers [tw] OR datasets [tw] OR trials [tiab] OR meta-analy* [tw] OR (clinical [tiab] AND studies [tiab]) OR treatment outcome [mh] OR treatment outcome [tw] OR pmcbook)) NOT (letter [pt] OR newspaper article [pt])
