# Supplementary material for: Protein intake and risk of urolithiasis and kidney diseases: an umbrella review of systematic reviews for the evidence-based guideline of the German Nutrition Society
Source: Eur J Nutr. 2023 May 3;62(5):1957–75. doi: 10.1007/s00394-023-03143-7 (PMC10349749; doi:10.1007/s00394-023-03143-7)
Supplement: Supplementary file 6 — Supplementary file6 (DOCX 16 KB) [file 394_2023_3143_MOESM6_ESM.docx]

**Supplementary Material S6:** List of excluded articles.

| **Reference** | **Reason for exclusion** |
| --- | --- |
| Barghouthy Y, Corrales M, Somani B (2021) The Relationship between Modern Fad Diets and Kidney Stone Disease: A Systematic Review of Literature. Nutrients 13. https://doi.org/10.3390/nu13124270 | Irrelevant study type |
| Eslami O, Shidfar F (2018) Dairy products and chronic kidney disease: protective or harmful? asystematic review of prospective cohort studies. Nutrition 55-56:21–28. https://doi.org/10.1016/j.nut.2018.03.047 | Relevant diet-disease relationship not investigated |
| Ko GJ, Obi Y, Tortorici AR et al. (2017) Dietary protein intake and chronic kidney disease. Curr Opin Clin Nutr Metab Care 20:77–85. https://doi.org/10.1097/MCO.0000000000000342 | Irrelevant study type |
| Major TJ, Topless RK, Dalbeth N et al. (2018) Evaluation of the diet wide contribution to serum urate levels: meta-analysis of population based cohorts. BMJ 363:k3951. https://doi.org/10.1136/bmj.k3951 | Relevant diet-disease relationship not investigated |
| Pedro RN, Aslam AU, Bello JO et al. (2020) Nutrients, vitamins, probiotics and herbal products: an update of their role in urolithogenesis. Urolithiasis 48:285–301. https://doi.org/10.1007/s00240-020-01182-x | Irrelevant study type |
| Quintela BCSF, Carioca AAF, Oliveira JGR de et al. (2021) Dietary patterns and chronic kidney disease outcomes: A systematic review. Nephrology (Carlton) 26:603–612. https://doi.org/10.1111/nep.13883 | Relevant diet-disease relationship not investigated |
| Shaughnessy AF (2013) Kidney stones: prevention options. Am Fam  Physician 88:609–610 | Irrelevant study type |
| Vasconcelos Q, Bachur TP, Aragão GF (2021) Whey protein  supplementation and its potentially adverse effects on health: a systematic  review. Appl Physiol Nutr Metab 46. https://doi.org/10.1139/apnm-2020-  0370 | Irrelevant study type |
| Yan B, Su X, Xu B et al. (2018) Effect of diet protein restriction on progression of chronic kidney disease: a systematic review and meta-analysis. PLoS One 13:e0206134. https://doi.org/10.1371/journal.pone.0206134 | Irrelevant population |
| Yuan S, Sun J, Lu Y et al. (2022) Health effects of milk consumption: phenome-wide Mendelian randomization study. BMC Med 20:455. https://doi.org/10.1186/s12916-022-02658-w | Relevant diet-disease relationship not investigated |
